# *Gemella haemolysans* inhibits the growth of the periodontal pathogen *Porphyromonas gingivalis*

**DOI:** 10.1038/s41598-021-91267-3

**Published:** 2021-06-03

**Authors:** Tomohiro Miyoshi, Shogo Oge, Satoshi Nakata, Yuji Ueno, Hidehiko Ukita, Reiko Kousaka, Yuki Miura, Nobuo Yoshinari, Akihiro Yoshida

**Affiliations:** 1 Department of Oral Microbiology, Matsumoto Dental University, 1780 Gobara Hirooka, Shiojiri, Nagano 399-0781 Japan; 2 Department of Periodontology, Matsumoto Dental University, 1780 Gobara Hirooka, Shiojiri, Nagano 399-0781 Japan

**Keywords:** Symbiosis, Clinical microbiology

## Abstract

The oral microbiome plays an important role in the human microbial community and in maintaining the health of an individual. Imbalances in the oral microbiome may contribute to oral and systemic diseases. The progression of periodontal disease is closely related to the growth of bacteria, such as *Porphyromonas gingivalis*, in the oral cavity. However, the pathogen growth mechanism specific to periodontal disease remains unknown. This study aimed to identify bacteria associated with periodontal health by focusing on hemolytic bacteria. Unstimulated saliva samples were collected from ten periodontitis patients and five healthy subjects to detect and identify the presence of hemolytic bacteria. The saliva of healthy subjects contained a higher proportion of *G. haemolysans* than saliva samples from patients with periodontitis. Growth inhibition assays indicated that the protein components contained in the culture supernatant of *G. haemolysans* directly suppressed the growth of *P. gingivalis*. This study shows that the presence of *G. haemolysans* in saliva is associated with periodontal health and that it inhibits the growth of *P. gingivalis* in vitro.

## Introduction

Periodontal disease is one of the most common chronic diseases in humans, with a very high prevalence among adults across the world^1–5^. It has increased the disease burden and has become an important public health concern in the aging global population. Studies on pathogens and inflammation in periodontal disease have attracted attention in the fields of medicine and dentistry owing to the potential influence of periodontitis on the initiation and progression of a variety of systemic diseases. Several studies have provided evidence linking periodontal disease to various non-oral systemic diseases, including cancer, neurodegenerative disease, diabetes, respiratory tract infections, adverse pregnancy outcomes, and cardiovascular disease^6–10^.

The pathological process of periodontitis involves the destruction of the periodontal structures that support the teeth, which is a major cause of tooth loss^11^. The development and progression of this disease are associated with a multifactorial process that depends on the interaction between the host cells and microbes in the oral cavity^12^. *Porphyromonas gingivalis* is a Gram-negative anaerobic bacterium that colonizes dental plaque in the oral cavity and is one of the major pathogens directly responsible for the development of chronic periodontitis in humans^13^. Several virulence factors such as cysteine protease (Rgp and Kgp)^14,15^, fimbriae (FimA and Mfa1)^16,17^, lipopolysaccharide (LPS)^18,19^ and the capsule^20,21^ influence the activities of this bacterium. These surface components and secretory enzymes contribute to efficient growth, nutrient acquisition, colonization, and protection from host defense mechanisms. The high pathogenicity of *P. gingivalis* has been investigated not only in cell models in vitro but also in various animal models such as the monkey^22^, mouse^23,24^, rabbit^25^, rat^26^, and fly^27^. Recent studies have implicated *P. gingivalis* in the onset of different systemic pathologies, including rheumatoid arthritis^28,29^, cardiovascular pathologies^30,31^, and neurodegenerative pathologies^32,33^.

The oral cavity harbors one of the most diverse microbiomes in the human body with genetic signatures of more than seven hundred bacteria^34,35^. This microbiome is constructed by reciprocal interactions between different bacterial communities and between the bacteria and the host environment^12^. Any disruption of the equilibrium of the oral microbiome allows for the emergence of disease-promoting bacteria, which cause conditions such as gingival inflammation and periodontitis. *Gemella* is a genus of Gram-positive facultatively anaerobic bacteria that is indigenous in the oral cavity and has hemolytic activity^36^. Metagenomic analysis of subgingival plaque samples showed that levels of *Gemella* spp. or *Gemella haemolysans* were higher in healthy controls than patients with periodontitis^37–39^. It has been reported that *G. haemolysans* levels increased after periodontal therapy in subgingival plaques of periodontitis patients^40^. On the other hand, there was no change in the prevalence of this bacterium in the mesial sulcus of teeth of periodontal subjects as compared with healthy controls^41^. *G. haemolysans* has been described as an early colonizer of oral biofilms and is part of the symbiotic microbial flora^42^. Outside of the oral cavity, this species is associated with opportunistic infections in immunocompromised patients and causes infectious endocarditis^43,44^.

Patients with periodontal disease often have bleeding gums. However, it is unclear whether there is a relationship between periodontal disease and hemolytic bacteria. Therefore, the aim of this study was to identify the hemolytic bacteria associated with periodontal health. Several hemolytic bacteria were identified in the saliva of patients with periodontal disease and healthy subjects. Sequence analysis of the 16S ribosomal RNA (rRNA) gene showed that the genus *Gemella* accounted for a large proportion of the hemolytic bacteria in saliva. The three *Gemella* species detected in saliva were *Gemella sanguinis*, *Gemella haemolysans*, and *Gemella morbillorum*. *G. haemolysans* levels were elevated in the oral cavities of healthy subjects compared to those in periodontal disease patients. The proteins secreted by *G. haemolysans* directly suppressed the growth of *P. gingivalis*, suggesting that *G. haemolysans* is associated with a healthy oral environment by suppressing the growth of *P. gingivalis*.

## Results

### Distribution of volunteers

Saliva samples from 15 volunteers at the Matsumoto Dental University Hospital were obtained to examine the differences in salivary microbiomes between periodontal patients and healthy subjects. The subjects were divided into two groups based on the presence (periodontal pocket depth, ≥ 4 mm; n = 10) or absence (n = 5) of periodontal disease. The average age of the 10 periodontal disease patients was 57.5 years (range, 35–69 years) and that of the 5 healthy subjects was 55.6 years (range, 49–64 years). No statistically significant difference in the age was noted between the periodontal disease patients and healthy subjects. The demographic and clinical parameters of the subjects in this study are summarized in Table 1.

**Table 1:** Summary of volunteers and category.

Variables	Mean	
	Periodontal disease patients	Healthy subjects
Number	10	5
Age (SD)	57.5 (9.7)	55.6 (6.3)
Gender (female/male)	9/1	2/3
Probing depth (mm; deep sites)	≥ 4	< 4

### Hemolytic bacteria in the saliva of the healthy subjects and periodontal disease patients

**Figure 1:**
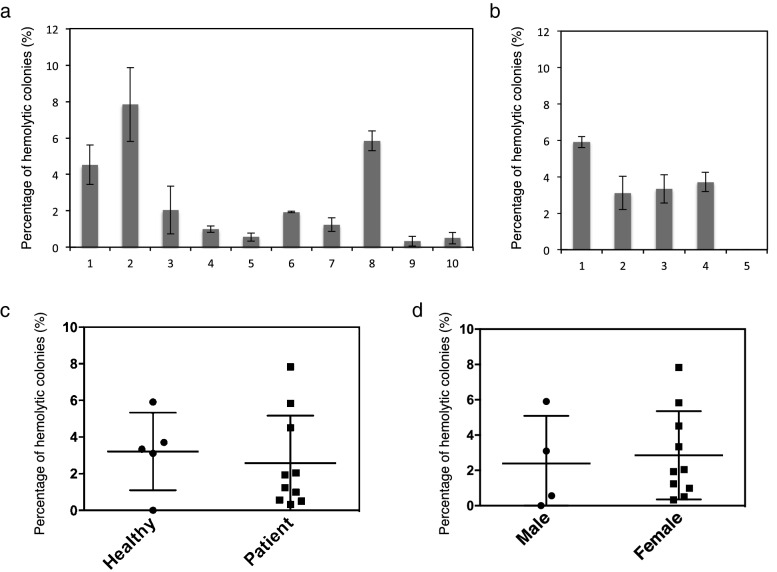
Measurement of hemolytic bacteria in human saliva. **(a,b)** Percentages of hemolytic bacterial cells in saliva from periodontal disease patients **(a)** or healthy subjects **(b)**. Individual saliva samples (n = 3) were spread on agar plates containing horse blood and incubated at 37 °C under anaerobic conditions. The numbers of hemolytic and non-hemolytic bacterial colonies were determined using the plate count technique. Error bars show the mean ± standard deviation (SD) values. **(c)** No significant differences in the percentage of hemolytic bacteria were observed between healthy subjects (black circles) and periodontal disease patients (black squares). Mann–Whitney U-test. Error bars show the mean ± standard error of mean (SEM) values. **(d)** No significant differences in the percentage of hemolytic bacteria in saliva were observed between male and female subjects. Mann–Whitney U-test. Error bars show the mean ± SEM.

A hemolytic activity assay of salivary bacteria was performed using horse blood agar plates to investigate the differences in the oral hemolytic bacteria between periodontal disease patients and healthy subjects. Some bacterial colonies in the saliva samples of the periodontitis patients and healthy subjects exhibited hemolytic activities (Fig. 1a,b). The average percentages of hemolytic colonies were 3.2% and 2.6% in the periodontitis patients and healthy subjects, respectively (Fig. 1c). Large variations in the proportions of hemolytic bacteria in the saliva were noted between individuals in the two groups (Fig. 1a,b); however, no significant differences were observed between the two groups of subjects (P = 0.59, Fig. 1c) or between males and females in this study (P = 0.67; Fig. 1d).

Sequence analysis of the 16S rRNA genes from hemolytic bacterial colonies was performed in two samples each from the periodontitis patients and healthy subjects. Highly hemolytic strains of *G. sanguinis*, *G. haemolysans,* and *Streptococcus mitis* were isolated from the saliva of the periodontal disease patients (Table 2). In addition to these bacteria, *Haemophilus parainfluenzae* and *Streptococcus australis* were isolated from healthy subjects. Furthermore, *G. sanguinis* and *G. haemolysans* accounted for 75% (15/20) of the isolated highly hemolytic bacteria, indicating that *Gemella* species represent a major proportion of the hemolytic bacteria present in the saliva of humans. Additionally, *G. morbillorum* and *Streptococcus cristatus* were identified as weakly hemolytic bacteria (Table 2).

**Table 2:** Bacterial species of colonies with hemolytic activity.

Strong hemolytic activity	Weak hemolytic activity
**Periodontal disease patient 1**	
1 *Gemella sanguinis *	1 *Gemella sanguinis*
2 *Gemella sanguinis *	2 *Streptococcus salivarius*
3 *Gemella sanguinis *	3 *Streptococcus salivarius*
4 *Gemella sanguinis *	4 *Streptococcus mitis*
5 *Streptococcus mitis*	
6 *Gemella haemolysans*	
**Periodontal disease patient 2**	
1 *Gemella sanguinis *	1 *Streptococcus australis*
2 *Gemella sanguinis *	2 *Delfita tsuruhatensis*
3 *Gemella sanguinis *	3 *Streptococcus australis*
4 *Streptococcus mitis *	4 *Streptococcus cristatus*
5 *Gemella sanguinis *	5 *Streptococcus australis*
6 *Gemella haemolysans*	
**Healthy subject 1**	
1 *Haemophilus parainfluenzae *	1 *Gemella morbillorum*
2 *Streptococcus australis *	2 *Streptococcus salivarius*
3 *Gemella haemolysans *	3 *Streptococcus mitis*
4 *Gemella sanguinis*	
**Healthy subject 2**	
1 *Gemella sanguinis *	1 *Haemophilus parainfluenzae*
2 *Streptococcus mitis *	2 *Streptococcus salivarius*
3 *Gemella haemolysans *	3 *Gemella haemolysans*
4 *Gemella sanguinis *	4 *Streptococcus mitis*
	5 *Gemella sanguinis*

### Ratio of *Gemella* species in the saliva of periodontal patients

**Figure 2:**
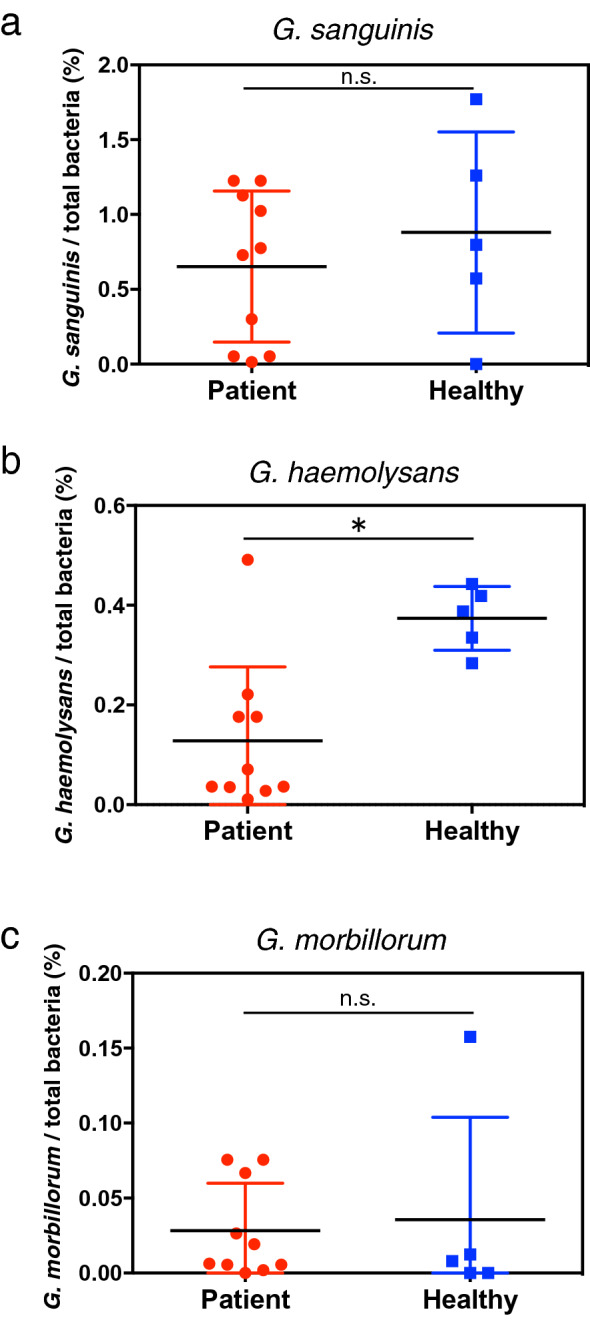
Levels of *G. haemolysans* in the saliva of healthy subjects and periodontitis patients. **(a–c)** Quantification of *Gemella* species in the saliva of healthy subjects and periodontal disease patients. The percentages of *G. sanguinis* **(a)**, *G. haemolysans* **(b)**, and *G. morbillorum* **(c)** among the total salivary bacteria were determined by quantitative PCR analysis using specific primers for the 16S rRNA gene. The results for samples from the periodontal disease patients and healthy subjects are shown as red circles and blue squares, respectively. All data were analyzed using a two-tailed Mann–Whitney U-test. ns, not significant. *P < 0.05. Error bars show the mean ± SEM.

*G. sanguinis*, *G. haemolysans,* and *G. morbillorum* were detected in human saliva (Table 2). qPCR was performed using specific primers for the 16S rRNA of each *Gemella* species to investigate whether the abundance ratios of the three *Gemella* species were different between the periodontitis patients and healthy subjects. *G. sanguinis* and *G. haemolysans* were more predominant than *G. morbillorum* in the saliva of the healthy subjects, (Supplementary Fig. 1). *G. haemolysans* was more prevalent in the saliva of the healthy subjects (Fig. 2b); however, there was no difference based on gender (Supplementary Fig. 2). No significant differences in the abundance ratios of *G. sanguinis* and *G. morbillorum* were observed between the two groups of subjects (Fig. 2a,c). These data provide a preliminary indication that high levels of *G. haemolysans* in saliva are associated with periodontal health.

### Growth inhibition of *P. gingivalis* by *G. haemolysans*

**Figure 3:**
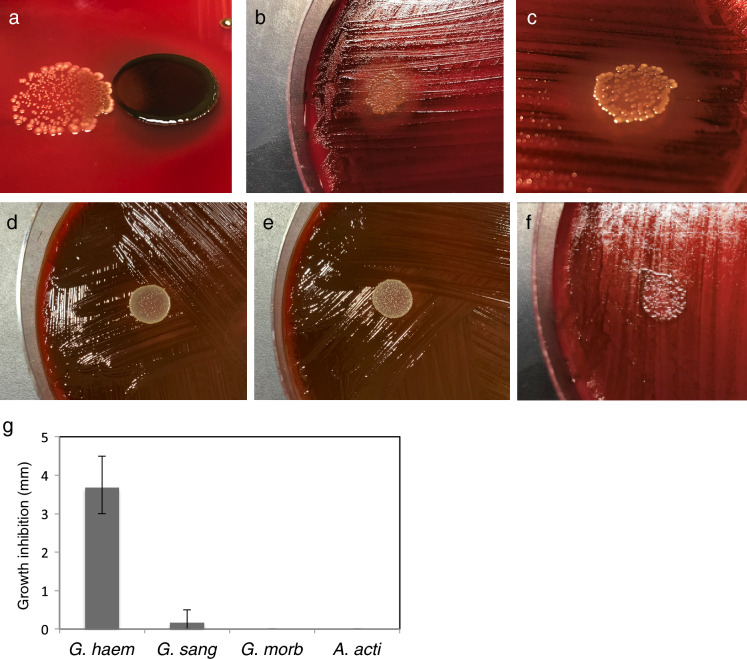
Effect of *Gemella species* on the growth of *P. gingivalis.* **(a)** Effect of *G. haemolysans* on the growth of *P. gingivalis*. Cultures of *G. haemolysans* and *P. gingivalis* were spotted adjacent to each other on a blood agar plate and incubated at 37 °C under anaerobic conditions. The white and black colored colonies indicate *G. haemolysans* and *P. gingivalis*, respectively. **(b–f)** Cultures of *G. haemolysans* **(b)**, *G. sanguinis* **(d)**, *G. morbillorum* **(e)** *and A. actinomycetemcomitans* **(f)** were each spotted on an agar plate spread with *P. gingivalis* and plates were incubated at 37 °C under anaerobic conditions. **(c)** The zoom image of **(b)** from another angle. **(g)** Quantification of the growth inhibition zone of *P. gingivalis* caused by *G. haemolysans (G. haem), G. sanguinis (G. sang), G. morbillorum (G. morb), A. actinomycetemcomitans (A. acti)*. Error bars show the mean ± SD. All results are representative of data generated in at least three independent experiments.

*P. gingivalis* is considered to be the main etiological agent in periodontal disease. Several studies have demonstrated the critical role of this bacterium in the pathogenesis of periodontal diseases^45,46^. To determine whether *G. haemolysans* is associated with the growth of periodontal pathogens, we investigated its effect on the growth of *P. gingivalis* using blood agar plates. The growth of *P. gingivalis* was inhibited in the region adjacent to colonies of *G. haemolysans* on agar plates (Fig. 3a). Furthermore, a growth inhibition zone for *P. gingivalis* was formed in regions close to the *G. haemolysans* colonies (Fig. 3b,c). On the other hand, *G. sanguinis*, *G. morbillorum* and *Aggregatibacter actinomycetemcomitans* did not affect the growth of *P. gingivalis* (Fig. 3d–g). These results indicate that *G. haemolysans* can directly suppress the growth of *P. gingivalis*.

### Inhibition of bacterial growth by *G. haemolysans*

**Figure 4:**
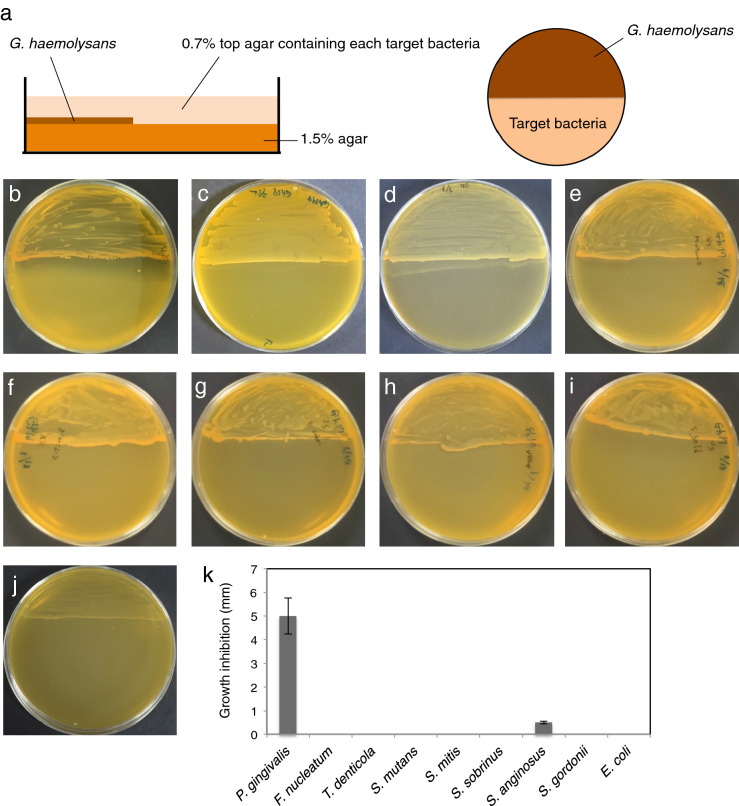
Target specificity of the growth inhibition activity of *G. haemolysans*. **(a)** Schematic depiction of the soft-agar overlay technique used to investigate the growth inhibition activity of *G. haemolysans*. **(b–j)** The soft 0.7% agar in the top layer was inoculated with the target bacterium i.e., *P. gingivalis* **(b)**, *F. nucleatum* **(c)**, *T. denticola* **(d)**, *S. mutans* **(e)**, *S. mitis* **(f)**, *S. sobrinus* **(g)**, *S. anginosus* **(h)**, *S. gordonii* **(i)** and *E. coli* **(j)**, while the 1.5% agar bottom layer was inoculated with *G. haemolysans*. **(k)** Quantification of the growth inhibition zone between *G. haemolysans* and each bacterium. Error bars show the mean ± SD. All results are representative of data generated in at least two independent experiments.

A soft-agar overlay assay was performed to determine whether the inhibitory activity of *G. haemolysans* was specific for *P. gingivalis*. The ability of *G. haemolysans* to suppress the growth of several different bacterial strains was examined within the top agar on plates with actively growing *G. haemolysans* (Fig. 4a). Each top agar contained nutrients required for the growth of each target bacterial strain. The presence of *G. haemolysans* caused a growth inhibition zone for *P. gingivalis* in the top agar, indicating a strong growth-suppression activity (Fig. 4b). To examine whether differences in the cell wall structure of the target bacteria affected the inhibitory activity of *G. haemolysans*, similar inhibition experiments were performed using several Gram-positive and Gram-negative bacteria. No growth-suppressing effects were observed on other periodontal pathogens (*Fusobacterium nucleatum* and *Treponema denticola*: Gram-negative), oral streptococci species (*S. mitis*, *S. mutans*, *S. sobrinus and S. gordonii*: Gram-positive), and enteric bacteria (*Escherichia coli*: Gram-negative), whereas a slight effect was detected on *Streptococcus anginosus* (Gram-positive; Fig. 4c–k). These data indicate that the growth suppression activity of *G. haemolysans* was particularly effective against *P. gingivalis*.

### Analysis of the culture supernatant of *G. haemolysans*

**Figure 5:**
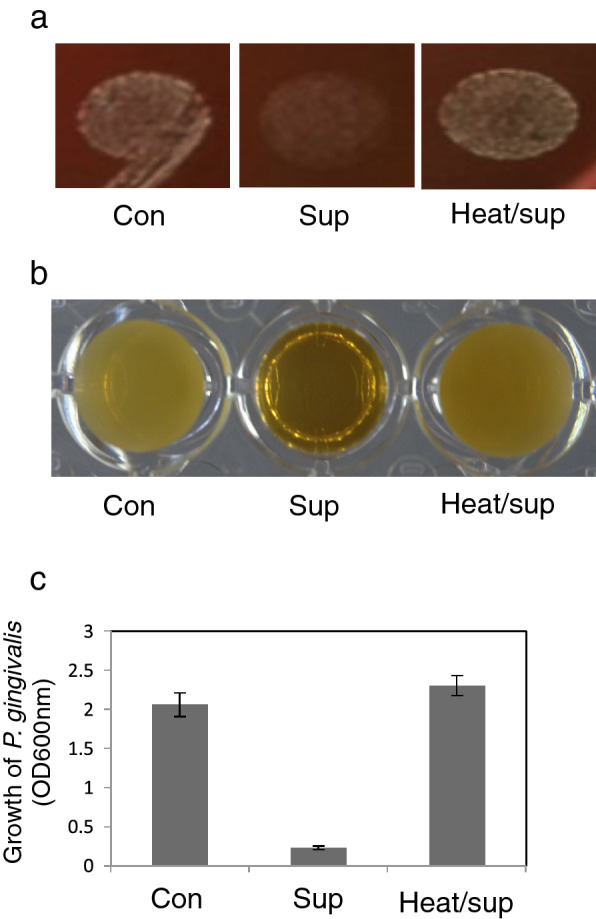
The cell culture supernatant of *G. haemolysans* exhibits the growth suppression ability against *P. gingivalis*. **(a)** *P. gingivalis* was cultured on blood agar containing sterile GAM medium (as a control, left panel), culture supernatant of *G. haemolysans* (middle panel), or heat-treated culture supernatant of *G. haemolysans* (right panel). **(b) ***P. gingivalis* was cultured in liquid medium containing PBS (as a control, left), culture supernatant of *G. haemolysans* (center), or heat-treated culture supernatant of *G. haemolysans* (right). **(c)** Quantification of the growth of *P. gingivalis* observed in **(b)**. The optical density was measured at 600 nm to determine the concentration of *P. gingivalis*. Error bars show the mean ± SD. All results are representative of data generated in at least three independent experiments.

A blood agar medium impregnated with *G. haemolysans* culture supernatant was prepared to investigate whether the inhibitory agent was present in the culture supernatant. The growth of *P. gingivalis* was clearly inhibited on agar medium containing the culture supernatant of *G. haemolysans* (Fig. 5a). In addition, the inhibitory activity was dramatically reduced using heat-treated supernatant, suggesting that this inhibitor is a heat-labile molecule (Fig. 5a). Next, we focused on the protein components contained in the culture supernatant of *G. haemolysans*. Secreted proteins in the culture supernatant of *G. haemolysans* were obtained by ammonium sulfate precipitation. After dialysis, the secreted proteins were used in the growth inhibition assay. *P. gingivalis* growth was barely visible in liquid medium containing the *G. haemolysans* secreted proteins compared to that in the control medium without the secreted proteins (Fig. 5b,c). In addition, the growth inhibition of *P. gingivalis* was dramatically reduced following heat treatment of the secreted proteins (Fig. 5b,c). These results suggest that one or more proteins secreted by *G. haemolysans* directly inhibited the growth of *P. gingivalis*.

## Discussion

In this study, we examined bacteria in the saliva of patients with periodontal disease and healthy controls. Saliva can be collected non-invasively, repeatedly and without trained personnel. Since the salivary microbiota are generally considered to reflect the overall periodontal condition in patients with periodontitis^47^, saliva is a promising diagnostic body fluid for clinical use in dentistry. In fact, research with oral plaque has shown that *G. haemolysans* levels increase after therapy for periodontal disease^40^, which is consistent with the high proportion of *G. haemolysans* in saliva samples from healthy subjects in our study.

The Human Oral Microbiome Database (https://www.homd.org) has registered four *Gemella* species with the scientific names *G. haemolysans*, *G. morbillorum*, *G. sanguinis* and *G. bergeri*. *G. haemolysans* was first described as *Neisseria haemolysans*^48^ because the bacterium was thought to be a Gram-negative aerobic diplococcus that causes hemolysis on rabbit blood agar. However, this organism did not possess the typical bacteriological characteristics of the genus *Neisseria*, such as catalase, cytochrome oxidase, and peroxidase activities^49^. For these reasons, a new genus was established. Likewise, *G. morbillorum* was previously identified as a *Peptostreptococcus* species. Analysis of the 16S rRNA gene sequence showed that *G. haemolysans* is more similar to *G. sanguinis* and considerably different from *G. bergeri*^50,51^.

Strong hemolytic activity is proposed as a leading characteristic of *G. haemolysans.* It distinguishes these bacteria from *G. morbillorum*^52^, which generally presents with no or weak hemolytic activity (α-hemolysis). In some cases, *G. sanguinis*^53^ and *G. bergeri*^50^ exhibit strong hemolytic activity depending on the blood and components of the animal species used in the medium. Furthermore, *G. bergeri* has not been detected in the microbiome of oral plaques using 16S rRNA gene sequencing, indicating the rarity of this species in the oral cavity^54^. In the current study, three *Gemella* species with hemolytic activity (*G. sanguinis*, *G. haemolysans*, and *G. morbillorum*) were detected in human saliva (Table 2). *G. bergeri* may have remained undetected as a hemolytic colony in the current study due to the aforementioned reasons.

The levels of *G. haemolysans*, but not *G. sanguinis* or *G. morbillorum*, were significantly elevated in the saliva of healthy subjects when compared to those in the periodontitis patients, suggesting that *G. haemolysans* plays a different role in the oral cavity than *G. sanguinis* and *G. morbillorum* (Fig. 2). A previous biochemical analysis showed that *G. haemolysans* could be distinguished from *G. sanguinis* and *G. morbillorum* due to its failure to produce acid from mannitol and sorbitol^55^; furthermore, *G. sanguinis* differs from *G. morbillorum* by producing alkaline phosphatase^55^. *G. bergeri* differs from *G. haemolysans*, *G. sanguinis*, and *G. morbillorum* by failing to produce acid from maltose and sucrose^50^. In an analysis of the zinc metalloproteinase (Zmp) protein family, *G. haemolysans* was found to possess all of the Zmp components (IgA, ZmpC, and ZmpD), except for ZmpB; in contrast, *G. sanguinis* and *G. morbillorum* carried only ZmpB, whereas *G. bergeri* did not possess any Zmp homolog^56^. These results highlight some of the unique characteristics of *G. haemolysans* in addition to the inhibitory activity against *P. gingivalis* that is not found in *G. sanguinis* and *G. morbillorum* (Fig. 3). However, further biochemical and genomic analyses need to be conducted to fully understand the role of the *Gemella* genus in the oral cavity.

The normal microbiome prevents the colonization of pathogens by competing for attachment sites or essential nutrients. Previous studies targeting *P. gingivalis* showed that the *S. intermedius* extracellular protein arginine deiminase induced the down-regulation of the *fimA*, and *mfa1* genes, which encode the major subunits of fimbriae^57,58^. Thus, this protein abolished biofilm formation without affecting the growth rate of *P. gingivalis*. On the other hand, *Bifidobacteria* have been reported to inhibit the growth of *P. gingivalis*^59^; this effect was thought to be caused by a decrease in the pH due to acid production, nutrient competition, and the action of inhibitor molecules. *G. haemolysans* can produce acid, similar to *Bifidobacterium*^60^. In the current study, the effect of a decrease in pH in the *P. gingivalis* growth inhibition assay was resolved by changing the buffer using a dialysis membrane, and nutrient competition was ruled out by using an extracellular fraction without live bacteria (Fig. 5b). These results clearly showed that the components of *G. haemolysans* in the culture supernatant inhibited the growth of *P. gingivalis*. In addition, we presumed that the component(s) responsible for this activity would be a protein because it could be concentrated via ammonium sulfate precipitation and inactivated by heat treatment. However, the protein involved in inhibiting the growth of *P. gingivalis* has not been identified so far; additional studies are needed to determine this in the future.

Periodontal disease is one of the most common chronic inflammatory diseases^1–5^. Many studies have shown the association of several bacterial pathogens with this disease^61^. Conversely, little attention has been paid to the identification of health-associated and potentially beneficial bacterial species in the oral cavity. Probiotics technology is a groundbreaking approach to maintaining health by using beneficial bacteria that can support the natural defense system against pathogens^62–64^. Probiotics have emerged as an attractive oral medicine and the use of these agents is increasing^65–67^. In the present study, the symbiotic bacterium *G. haemolysans* suppressed the growth of *P. gingivalis*. This data presents *G. haemolysans* as a novel probiotic candidate for treating periodontal disease. Furthermore, secreted protein(s) from *G. haemolysans* that function similar to a bacteriocin might be used for the treatment of periodontal disease as a *P. gingivalis*–targeting drug. Nonetheless, it is essential to develop an understanding of the broad range of environmental changes that would be caused by the ingestion of *G. haemolysans* in the oral cavity and their long-term effects on oral diseases and health.

This study proposes a novel role for indigenous bacteria in controlling periodontal pathogens. However, the results are limited to the analysis of two bacterial strains using an artificial medium; hence the in vivo effect of *G. haemolysans* on the oral flora remains unclear. Additional studies are required to understand the relationship between *G. haemolysans* and periodontal disease in the oral cavity in more detail.

## Conclusion

In summary, the findings of this study showed that the saliva of healthy subjects contained a higher proportion of *G. haemolysans* than that of patients with periodontitis. Growth inhibition assays indicated that the protein components contained in the culture supernatant of *G. haemolysans* directly suppressed the growth of *P. gingivalis*. These findings show that the presence of *G. haemolysans* in saliva is associated with oral health and that it inhibits the growth of *P. gingivalis* in vitro, which may prove useful during the development of therapies for this disease in the future.

## Materials and methods

### Ethics

The study was reviewed and approved by the research ethics committee of the Matsumoto Dental University (#0217) and all methods were carried out in accordance with relevant guidelines and regulations. All study subjects signed informed consent prior to participating.

### Collection of saliva samples

Saliva samples were collected without stimulation from 15 volunteers at the Matsumoto Dental University Hospital in Japan. Data concerning the age, sex, and pocket depth, and location of the subjects were recorded during sample collection. Those with a periodontal pocket depth of ≥ 4 mm were categorized as patients with periodontal disease (n = 10) and the remaining 5 with depths of < 4 mm were grouped as healthy subjects.

### Detection and identification of hemolytic bacteria in saliva

Saliva samples were dispersed by vortexing followed by a tenfold serial dilution with phosphate-buffered saline (PBS). Aliquots of 100 µl of each dilution were plated on TS blood agar (Trypticase Soy Broth; 5% horse blood and 1.5% agar) and incubated for 48 h at 37 °C in an anaerobic chamber (Anaerobox, Hirasawa, Tokyo, Japan) containing 85% N_2_, 10% H_2_, and 5% CO_2_. The colonies on the agar medium were clearly distinguished into hemolytic and non-hemolytic bacteria, and the numbers of each colony type were counted.

### 16S ribosomal RNA gene sequencing

Bacterial genomic DNA was extracted from each hemolytic colony using a heat extraction method^68^. The 16S rRNA genes of each hemolytic bacterium were amplified using KAPA HiFi HS ReadyMix (Kapa Biosystems), according to the manufacturer’s instructions. A polymerase chain reaction (PCR) amplification was performed using the following universal bacterial primers: 16S_27f (5′-AGA GTT TGA TCC TGG CTC AG-3′) and 16S_1492r (5′-GGT TAC CTT GTT ACG ACT T -3′)^69^. The PCR products were gel purified using the FastGene Gel/PCR Extraction Kit (Nippon Genetics, Japan) and the sequences were confirmed via a commercial sequencing service (Fasmac, Kanagawa, Japan). The sequences of the PCR products were compared with known 16S rRNA gene sequences in GenBank, and the hemolytic bacterial species were identified.

### Quantification of each *Gemella* species in saliva by quantitative PCR

The extraction of bacterial DNA from saliva was performed using MORA-EXTRACT (Kyokuto Seiyaku, Tokyo, Japan) according to the manufacturer’s instructions. To compare the amount of *Gemella* species and total bacteria in saliva, quantitative real-time PCR (qRT-PCR) was performed using a Fast SYBR Green Master Mix (Applied Biosystems) according to the manufacturer’s instructions. The universal bacterial primers for the 16S rRNA gene in total bacteria (16S_27f; 5′-AGA GTT TGA TCC TGG CTC AG-3′, 16S_350r; 5′-CTG CTG CCT CCC GTA G -3′) and specific primers for the 16S rRNA genes in *G. sanguinis* (Gs153-176f; 5′-ATA ACA GCA TAA ATC GCA TGA TAT-3′, Gs455-478r; 5′-TGG TTA GGT ACC GTC TCT ACT GTA-3′), *G. haemolysans* (Gh170-189f; 5′-CAG CAT TAA CTG CAT GGT TG-3′, Gh467-489 5′-GGT TAG GTA CCG TCT CTA CTG TG-3′) and *G. morbillorum* (Gm165-188; 5′-ATA ACA GTA TTT CTC GCA TGA GAG-3′, Gm466-488;5′-GGT TAG GTA CCG TCT CTT ACA TG-3′) were used. The PCR cycling conditions were as follows: 95 °C for 30 s followed by 40 cycles of 95 °C for 5 s and 58 °C for 1 min (Takara Thermal Cycler Dice Real-Time System II). DNA melting curves were created to check for the presence of only one amplification fragment. The specificity of each primer set was confirmed by analyzing the PCR products by agarose gel electrophoresis (Supplementary Figs. 3 and 4). The relative amounts of total bacteria and *Gemella* species in saliva were calculated using the comparative CT method^70^.

### Growth competition assay

To evaluate the ability of *G. haemolysans* to inhibit *P. gingivalis* growth, a growth competition assay was performed by culturing the two types of bacteria in close proximity to each other on blood agar plates. Strains of *G. haemolysans* (clinical isolates obtained in this study), *G. sanguinis* ATCC700632, *G. morbillorum* ATCC27824, *P. gingivalis* W83, and *A. actinomycetemcomitans* NK1651 were each cultured in Gifu anaerobic medium (GAM) containing hemin (5 µg/ml) and menadione (0.5 µg/ml) at 37 °C under anaerobic conditions containing 85% N_2_, 10% H_2_, and 5% CO_2_. After reaching the late stationary phase (approximately 48 h), 10 µl of *G. haemolysans* culture was spotted onto a blood agar plate and the *P. gingivalis* culture was spotted adjacent to it; the plate was incubated for 4 days at 37 °C under anaerobic conditions. In addition, the *P. gingivalis* culture was spread thinly on blood agar plates using a swab. After drying the plate, 10 µl of each culture of *G. haemolysans*, *G. sanguinis, G. morbillorum* and *A. actinomycetemcomitans* was spotted onto the blood agar plate, which was incubated for 4 days at 37 °C under anaerobic conditions. The inhibitory activity was detected by measuring the growth inhibition zone of *P. gingivalis* on the agar plates.

The growth inhibition assay for bacteria that grow in special media was performed using the soft-agar overlay technique^71,72^. *G. haemolysans* was spread onto half the area of a GAM agar (1.5%) plate and cultured for 2 days at 37 °C under anaerobic conditions. Next, a soft-agar (0.7%) medium (5 ml, 50 °C) containing each target bacterial strain (0.3 ml) was overlaid on the 1.5% agar plate and incubated for 48 h at 37 °C under anaerobic conditions. A modified GAM broth (Nissui) containing thiamin (2 µg/ml) and FBS (5%) was used as a soft-agar medium for *T. denticola* JCM8225, whereas GAM broth containing hemin (5 µg/ml) and menadione (0.5 µg/ml) was used for *F. nucleatum* JCM6328, *S. anginosus* NCTC10713, *S. mitis* ATCC9811, *S. mutans* MT8148, *S. gordonii* ATCC35105*, S. sobrinus* GTC278, and *E. coli* BL21. The growth inhibition activity of *G. haemolysans* for each bacterial strain was detected by measuring the zone of growth inhibition on the top agar.

### Growth inhibition assay using bacterial culture supernatant

An assay was carried out using the bacterial culture supernatant of *G. haemolysans* to investigate the mechanism by which it inhibited the growth of *P. gingivalis*. The bacterial culture supernatant was obtained by culturing *G. haemolysans* in GAM medium for 48 h at 37 °C under anaerobic conditions and filtering it using a vacuum filtration system (rapid-Filtermax 0.2 µm, TPP, Switzerland). A *P. gingivalis* culture (10 µl) was spotted onto a blood agar plate soaked with the supernatant (2 ml) and incubated for 48 h at 37 °C under anaerobic conditions. The same process was performed for the heat-treated (at 90 °C for 5 min) supernatant and sterile GAM medium as a control. The growth rate of *P. gingivalis* on the blood agar was evaluated.

Furthermore, the inhibition activity was confirmed in liquid cultures. Proteins in the culture supernatant of *G. haemolysans* were obtained via ammonium sulfate precipitation. The 20-fold concentrated sample was dialyzed (VISKASE, MEMBRA-CEL dialysis membranes, MWCO: 14,000) in a buffer (20 mM Tris–HCl pH7.5, 150 mM NaCl) for 3 h, and a portion of the sample was incubated for 5 min at 90 °C. Each sample (20 µl) was filtered using MILLEX GV 0.22 µm filters (Millipore) and mixed with *P. gingivalis* (180 µl; OD at 600 nm, 0.04) diluted in GAM medium containing hemin (5 ug/ml) and menadione (0.5 µg/ml), and incubated in a 96 well culture plate (CELLSTAR, Greiner Bio-one, Australia) for 48 h at 37 °C under anaerobic conditions. The inhibition activity was evaluated by measuring the optical density at 600 nm (BioPhotometer-D30, Eppendorf, Germany).

## Supplementary Information

Supplementary Figures.
