# Supplementary material for: Gemella haemolysans inhibits the growth of the periodontal pathogen Porphyromonas gingivalis
Source: Sci Rep. 2021 Jun 3;11:11742. doi: 10.1038/s41598-021-91267-3 (PMC8175725; doi:10.1038/s41598-021-91267-3)
Supplement: Supplementary file 1 — Supplementary Figures. [file 41598_2021_91267_MOESM1_ESM.pdf]

Supplementary Data

Gemella haemolysans inhibits the growth of the periodontal pathogen *Porphyromonas gingivalis*

Tomohiro Miyoshi[a*], Shogo Oge[a], Satoshi Nakata[a], Yuji Ueno[a], Hidehiko Ukita[a], Reiko Kousaka[a], Yuki Miura[a], Nobuo Yoshinari[b], and Akihiro Yoshida[a*].

[a]Department of Oral microbiology, [b]Department of Periodontology, Matsumoto Dental University, 1780 Gobara Hirooka, Shiojiri, Nagano, 399-0781, Japan

*Corresponding authors:

Tomohiro Miyoshi (e-mail: tomohiro.miyoshi@mdu.ac.jp)

Akihiro Yoshida (e-mail: akihiro.yoshida@mdu.ac.jp)

Supplementary Figures 1 to 4

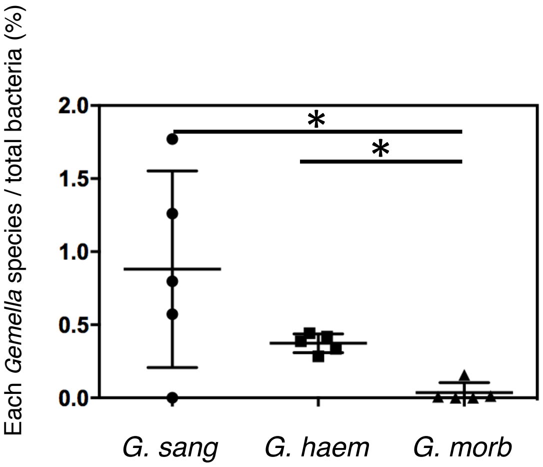

Supplementary figure 1. Comparison of the abundances of *Gemella* species in saliva of healthy subjects. The percentages of *G. sanguinis*, *G. haemolysans*, and *G. morbillorum* among total salivary bacteria were determined by quantitative PCR analysis using specific primers for the 16S rRNA gene. *G. sanguinis*, *G. haemolysans*, and *G. morbillorum* are shown as *G. sang*, *G. haem*, and *G. morb*, respectively. Data were analyzed using the two-tailed Mann-Whitney U-test. *P < 0.05. Error bars indicate the mean ± SEM.

Supplementary Figure 1

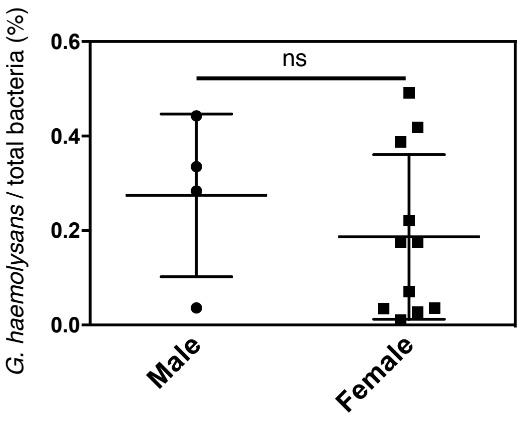

Supplementary figure 2. Comparison of the abundance of *Gemella haemolysans* in saliva between male and female subjects. The percentage of *G. haemolysans* among total salivary bacteria was determined by quantitative PCR analysis using specific primers for the 16S rRNA gene. Data were analyzed using the two-tailed Mann-Whitney U-test. ns, not significant. Error bars indicate the mean ± SEM.

Supplementary Figure 2

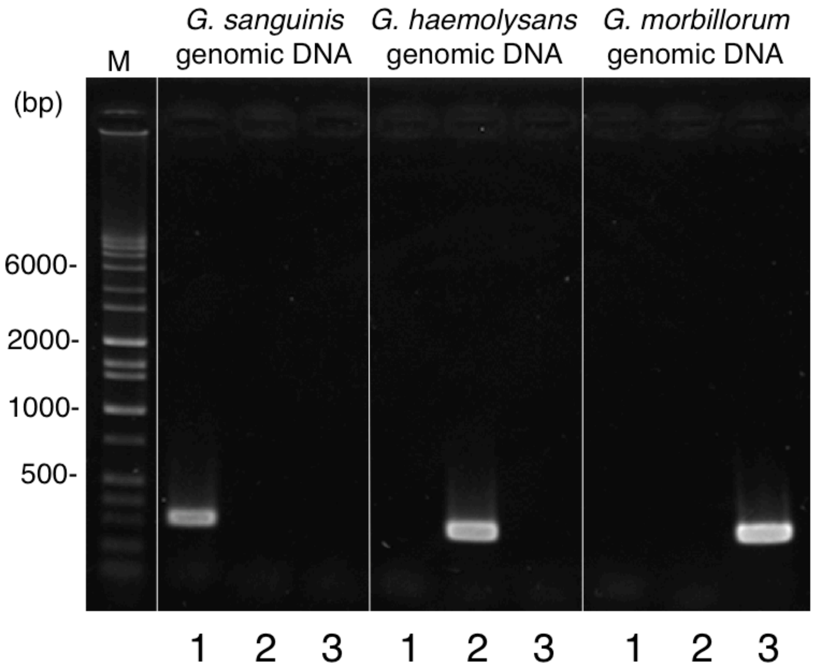

Supplementary figure 3. Analysis of primer specificity for genomic DNA of *G. sanguinis, G. haemolysans* and *G. morbillorum*. The specificity of each primer set for the 16S rRNA gene of *G. sanguinis* (lane 1), *G. haemolysans* (lane 2) and *G. morbillorum* (lane 3) was tested by PCR using genomic DNA. PCR reactions were performed under the same conditions as qPCR and the PCR products were analyzed by 1.5% agarose gel electrophoresis. The results were visualized with ethidium bromide. M: DNA marker.

Supplementary Figure 3

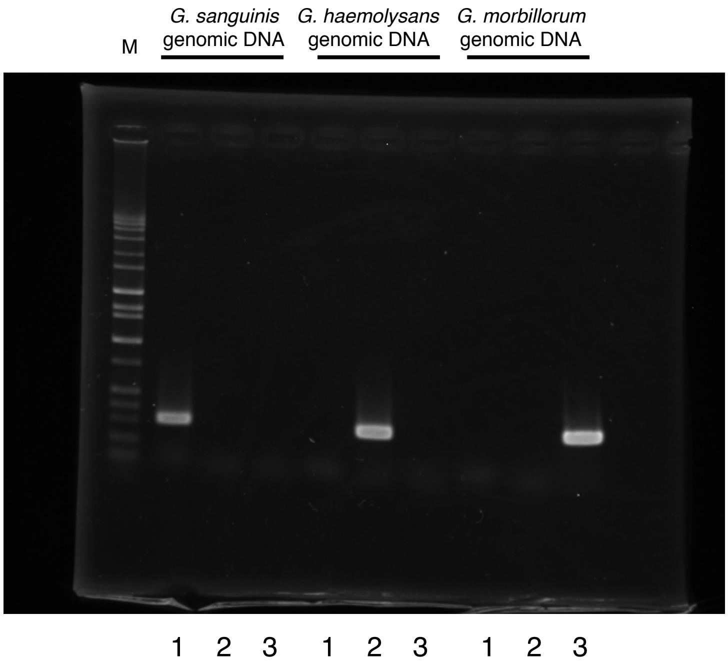

Supplementary figure 4. The original image of Supplementary figure 3.

Supplementary Figure 4
